# Prediction and impact of high burden of ventricular pacing in patients with pacemaker after transcatheter aortic valve replacement

**DOI:** 10.1016/j.hroo.2025.05.024

**Published:** 2025-05-27

**Authors:** Frédéric Anselme, Iliès Jaballah, Arnaud Savoure, Raphaël Al Hamoud, Charles Fauvel, Eric Durand, Hélène Eltchaninoff, Corentin Chaumont

**Affiliations:** 1 Department of Cardiology, Rouen University Hospital, UNIROUEN, INSERM U1096, Rouen, France; 2 Department of Cardiology, Rouen University Hospital, Rouen, France

**Keywords:** TAVR, TAVI, Pacemaker, Right ventricular pacing burden, Pacing-induced cardiomyopathy

## Abstract

**Background:**

A high burden of right ventricular pacing (RVP) increases the risk of hospitalization because of heart failure. Data on predictive factors for high burden of RVP in patients with permanent pacemaker implantation (PPI) after transcatheter aortic valve replacement (TAVR) are limited.

**Objective:**

This study aimed to identify predictors of high RVP burden in patients with current indications for PPI after TAVR.

**Methods:**

We included consecutive patients who underwent PPI after TAVR between 2013 and 2023 at our institution. Dual-chamber pacemakers were programmed with an algorithm favoring spontaneous atrioventricular (AV) conduction. High burden of RVP was defined as a pacing percentage of > 20% (> 20% ventricular pacing [VP]) at 3- to 12-month follow-up.

**Results:**

Among 193 patients included, 92 (47.7%) had > 20% VP at 3- to 12-month follow-up. Male gender (odds ratio [OR] 2.48, 95% confidence interval [CI] 1.31–4.67), permanent atrial fibrillation (OR 2.49, 95% CI 1.01–6.15), and high-degree AV block as the indication for PPI (OR 5.05, 95% CI 2.32–11.0) were independent predictors of > 20% VP. A H_2_AS risk score predicting > 20% VP was derived, including high-degree AV block (2 points), permanent atrial fibrillation (1 point), and male sex (1 point). A score of ≥ 3 identified a 68% prevalence of > 20% VP. Over a median follow-up of 27.7 months, > 20% VP was associated with a higher risk of all-cause mortality or heart failure hospitalization (hazard ratio 2.03, 95% CI 1.09–3.81, *P* = .03).

**Conclusion:**

A high RVP burden of > 20% can be anticipated using a readily available pre-PPI risk assessment. The H_2_AS risk score may assist clinicians in determining the most appropriate VP strategy for patients after TAVR with an indication for PPI.

**Key Findings**

▪ Among patients undergoing permanent pacemaker implantation after transcatheter aortic valve replacement, approximately 50% had a right ventricular pacing burden of > 20% at 3- to 12-month follow-up.
▪ Male sex, permanent atrial fibrillation, and high-degree atrioventricular block as the indication for permanent pacemaker implantation were independent predictors of a right ventricular pacing burden of > 20%.
▪ A high ventricular pacing burden (> 20%) was associated with an increased risk of all-cause mortality or heart failure hospitalization.

## Introduction

Since the first-in-human transcatheter aortic valve replacement (TAVR) by Cribier and colleagues^1^ in April 2002, the indications for TAVR have progressively expanded to younger, lower-risk patients with severe aortic stenosis.^2^ Despite advancements in procedural techniques and prosthetic valve design, cardiac conduction disorders remain one of the most frequent complications after TAVR. The reported incidence of periprocedural permanent pacemaker implantation (PPI) ranges from 6% to 35%, depending on the study and the valve type implanted.^3^ A high rate of right ventricular pacing (RVP) has been associated with an increased risk of hospitalization for heart failure (HF), owing to the development of pacing-induced cardiomyopathy (PICM).^4^, ^5^, ^6^ TAVR patients represent a vulnerable population with an inherently high risk of HF-related complications.^7^ Among patients requiring PPI after TAVR, the average ventricular pacing (VP) rate at 1 year was reported to be approximately 59%.^8^ However, a substantial proportion of these patients (25%) exhibit very low pacing rates (< 1%),^9^ whereas limited data are available on the proportion of patients with RVP rates exceeding 20%, a threshold associated with a heightened risk of PICM.^10^^,^^11^ To date, no study has attempted to identify predictors of a high RVP burden (>20%) in patients requiring PPI after TAVR. This parameter is gaining importance given that alternative pacing strategies, such as conduction system pacing (CSP) and leadless pacemakers, are now available. This study aimed to identify predictive factors of a high RVP burden (> 20%) in patients requiring PPI after TAVR to guide the selection of optimal pacing strategies.

## Methods

### Study population

This study included all consecutive patients who underwent PPI for cardiac conduction disturbances after TAVR between January 2013 and July 2023 at our institution. Patients were excluded if they were not followed up at our center or if they received CSP or bi-VP because the latter could have modified pacing programming and patients’ outcomes. Data were extracted from the ROUEN-TAVI registry, a prospective local database that includes all patients treated with TAVR for severe aortic stenosis at Rouen University Hospital. The database has been approved by the French ethics committee (2020-A00598-31). All patients provided a written informed consent for data collection and analysis. The research reported in this study adhered to the Declaration of Helsinki guidelines.

### Study protocol and definitions

All patients received an RVP lead. Dual-chamber pacemakers were all programmed with an algorithm favoring spontaneous atrioventricular (AV) conduction, except in cases of persistent complete AV block at discharge, where a tracking mode (DDD mode) was selected. At the routine 3-month pacemaker interrogation, the percentage of RVP was recorded, and AV conduction recovery was assessed. If AV conduction recovery was confirmed, pacemakers were reprogrammed to an algorithm favoring spontaneous AV conduction and the 1-year RVP percentage was used for the primary analysis. A high burden of RVP was defined as an RVP of > 20%. Patients were divided into 2 groups based on this threshold: ≤ 20% VP and >20% VP. The primary objective was to identify independent predictive factors of a high burden of RVP (> 20% VP). The secondary objective was to evaluate a composite endpoint of all-cause mortality or first HF hospitalization (HFH) over a 3-year follow-up period, stratified by RVP percentage. The individual components of the composite endpoint were also analyzed. HFH was defined as a hospital admission lasting at least 24 hours with a primary diagnosis of HF, new or worsening symptoms of HF at presentation, and initiation or intensification of HF-specific treatment.^12^

### Statistical analysis

Continuous variables were expressed as mean ± standard deviation for normally distributed data or as median and interquartile range (IQR) for non-normally distributed data. Categorical variables were expressed as counts and percentages. Comparisons between groups were performed using the chi-square test or Fisher’s exact test for categorical variables and Student’s *t* test or Mann–Whitney test for continuous variables. A logistic regression analysis was performed to identify predictive factors of RVP percentage of > 20%. Variables with a univariable *P* value of <.1 were entered in the multivariable model. Results were reported as odds ratios (ORs) with 95% confidence intervals (CIs). For the 3-year composite endpoint (all-cause mortality or first HFH), a Kaplan-Meier survival curve was plotted. Patients who did not experience the composite endpoint were censored as event-free at 3 years or at the time of their last clinical evaluation if the 3-year visit had not yet occurred. Cox proportional hazards regression analysis was used to identify predictive factors for the composite endpoint. Variables with a univariable *P* value of < .1 were included in the multivariable model. Results were reported as hazard ratios (HRs) with 95% CI. All statistical tests were 2 sided, with a *P* value of < .05 considered statistically significant. All analyses were performed using SPSS software (version 29.0; IBM Corporation, Armonk, NY).

## Results

### Baseline characteristics

**Figure 1:**
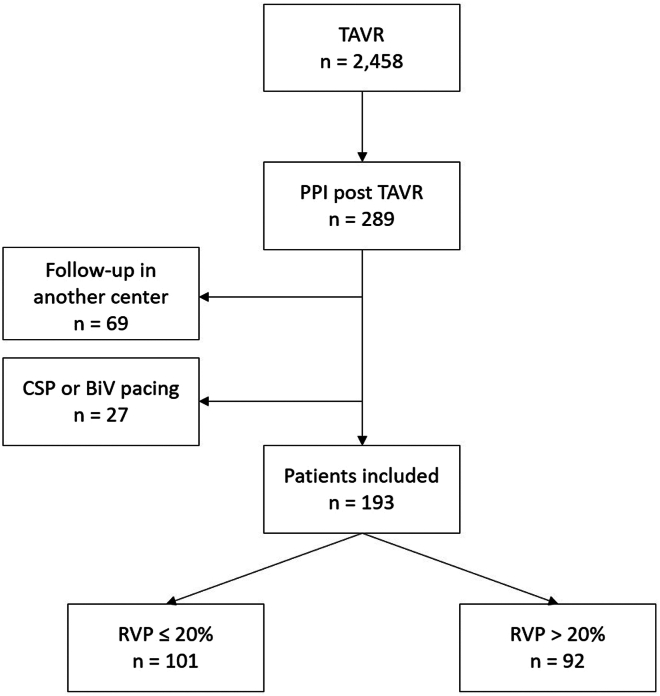
Study flowchart. BiV = biventricular; CSP = conduction system pacing; PPI = permanent pacemaker implantation; RVP = right ventricular pacing; TAVR = transcatheter aortic valve replacement.

From January 2013 to July 2023, 2458 consecutive patients underwent TAVR at our institution. Of these, 289 patients (11.8%) required PPI. Among these, 27 patients received a CSP lead or bi-VP, and 69 were followed up at other institutions for pacemaker management. These patients were excluded from the analysis (Figure 1). The final study population consisted of 193 patients (104 men [53.9%] with a mean age of 83.9 ± 6.6 years). Permanent atrial fibrillation (AF) was present in 33 patients (17.1%). A balloon-expandable valve was used in 132 patients (68.4%). The mean baseline left ventricular ejection fraction (LVEF) was 58.6% ± 12.7% (Table 1). Among the patients, 135 (69.9%) received PPI for high-degree AV block, 48 (24.9%) for left bundle branch block (LBBB) associated with an His-ventricular (HV) interval of > 70 ms, and 10 (5.2%) for other indications, including PR prolongation with an HV interval of > 70 ms (n = 7) or sinus node dysfunction (n = 3).

**Table 1:** Baseline characteristics

Characteristics	All patients (n = 193), mean ± SD or n (%)	RVP of ≤ 20% (n = 101), mean ± SD or n (%)	RVP of > 20% (n = 92), mean ± SD or n (%)	*P* value
Age (y)	83.9 ± 6.6	83.4 ± 7.3	84.5 ± 5.6	.2
Sex (male)	104 (53.9)	43	61	< .001
BMI (kg/m^2^)	26.6 ± 5.1	26.9 ± 5.4	26.2 ± 4.6	.3
Dyslipidemia	116 (60.1)	58	58	.4
Hypertension	147 (76.2)	75	72	.5
Diabetes	50 (25.9)	23	27	.3
Permanent AF	33 (17.1)	8	25	< .001
History of syncope	9 (4.7)	5	4	> .9
Coronary artery disease	71 (36.8)	37	34	> .9
Previous MI	15 (7.8)	6	9	.3
Previous PCI	48 (24.9)	25	23	> .9
Previous CABG	12 (6.2)	6	6	.9
Balloon-expandable	132 (68.4)	65	67	.2
Valve-in-valve implant	12 (6.2)	8	4	.4
LVEF (%)	58.6 ± 12.7	60.0 ± 13.2	57.2 ± 12.1	.2
Mean gradient (mm Hg)	46.1 ± 14.3	45.5 ± 14.7	46.7 ± 13.9	.5
LVEDD (mm)	51.2 ± 8.1	50.3 ± 8.0	52.0 ± 8.2	.2
LVESD (mm)	34.8 ± 8.1	33.8 ± 8.2	35.7 ± 8.1	.2
Respiratory failure	26 (13.5)	10	16	.1

AF = atrial fibrillation; BMI = body mass index; CABG = coronary artery bypass graft; LVEDD = left ventricular end-diastolic diameter; LVEF = left ventricular ejection fraction; LVESD = left ventricular end systolic diameter; MI = myocardial infarction; PCI = percutaneous coronary intervention; RVP = right ventricular pacing; SD = standard deviation.

### Pacemaker programming and VP burden

Among the study population, 47 patients (24.4%) received a single-chamber pacemaker, whereas 146 patients (75.6%) were implanted with a dual-chamber pacemaker. At the 3-month pacemaker interrogation, 55 of the 146 dual-chamber pacemakers (37.7%) were programmed in DDD mode, and 91 (62.3%) were programmed with an algorithm favoring spontaneous AV conduction. Among the patients in DDD mode, 12 of 55 (21.8%) demonstrated AV node conduction recovery and were subsequently reprogrammed. For these patients, the RVP percentage was reassessed at 1-year follow-up.

Based on the defined threshold, 92 patients (47.7%) were classified in the > 20% VP group, with 81 of them (88%) exhibiting an RVP burden exceeding 40%. All patients in the > 20% VP group continued to have a high RVP percentage at their last follow-up visit (median follow-up 25.8 months, IQR 12.8–36.6 months).

A total of 101 patients (52.3%) were in the ≤ 20% VP group. Most of these patients (89/101; 88.1%) maintained an RVP burden of ≤ 20% at their last follow-up (median follow-up 27.9 months, IQR 11.9–36.6 months).

No significant differences in age, BMI, cardiovascular risk factors, severity of aortic stenosis, LVEF, or valve type were observed between the 2 groups (Table 1).

### Predictive factors of > 20% VP

In the multivariable analysis, male gender (OR 2.48, 95% CI 1.31–4.67), permanent AF (OR 2.49, 95% CI 1.01–6.15), and high-degree AV block as the indication for PPI (OR 5.05, 95% CI 2.32–11.0) were identified as independent predictors of an RVP burden exceeding 20% (Table 2). Interestingly, preprocedural right bundle branch block, LBBB, and first-degree AV block were not associated with an increased likelihood of RVP of > 20%.

**Table 2:** Predictors of ventricular pacing of > 20% at 3- to 12-month follow-up

Variables	Univariable OR (95% CI)	*P* value	Multivariable OR (95% CI)	*P* value
Age (y)	1.03 (0.98–1.08)	.23		
Sex (male)	2.65 (1.48–4.77)	.001	2.48 (1.31–4.67)	.005
BMI (kg/m^2^)	0.97 (0.92–1.03)	.35		
Balloon-expandable	1.52 (0.81–2.86)	.19		
Valve-in-valve implant	0.56 (0.16–1.94)	.36		
LVEF	0.98 (0.96–1.01)	.16		
Respiratory failure	1.92 (0.82–4.47)	.13		
Pre-TAVR ECG characteristics				
RBBB	1.37 (0.70–2.70)	.37		
LBBB	1.12 (0.40–3.13)	.83		
PR of > 200 ms	1.34 (0.66–2.73)	.41		
Permanent atrial fibrillation	4.34 (1.84–10.21)	< .001	2.49 (1.01–6.15)	.048
Indication for pacemaker implantation				
High-degree AV block	6.41 (3.05–13.45)	<.001	5.05 (2.32–11.0)	<.001
LBBB with HV interval of > 70 ms	0.20 (0.09–0.44)	<.001		
Other∗	0.11 (0.01–0.91)	.04		

AV = atrioventricular; BMI = body mass index; CI = confidence interval; ECG = electrocardiogram; HV = His-ventricular; LBBB = left bundle branch block; LVEF = left ventricular ejection fraction; OR = odds ratio; RBBB = right bundle branch block; TAVR = transcatheter aortic valve replacement.

∗ Sinus node dysfunction or PR prolongation with HV interval of > 70 ms.

### Risk score predicting high burden of RVP

**Figure 2:**
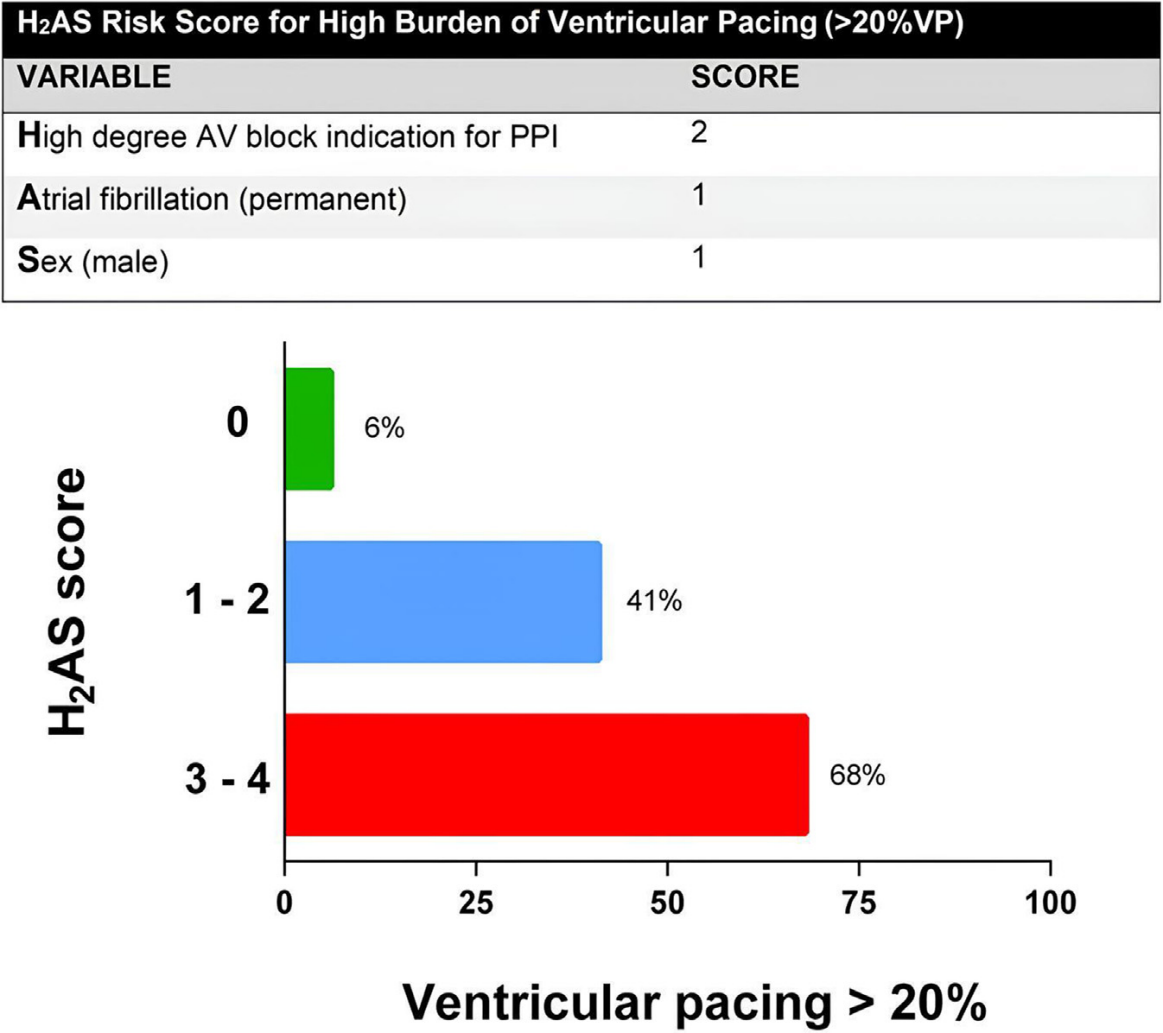
Percentage of patients with VP rate of > 20% according to H_2_AS score. AV = atrioventricular; PPI = permanent pacemaker implantation; RVP = right ventricular pacing; VP = ventricular pacing.

A risk score (H_2_AS score) was developed from the multivariable model to predict an RVP burden exceeding 20%. The score assigned 2 points for high-degree AV block as the indication for PPI, 1 point for permanent AF, and 1 point for male sex. The prevalence of RVP of >20% at 3- to 12-month follow-up increased with higher H_2_AS scores: 6% for a score of 0, 41% for a score of 1–2, and 68% for a score of ≥ 3 (Figure 2).

### Follow-up

**Figure 3:**
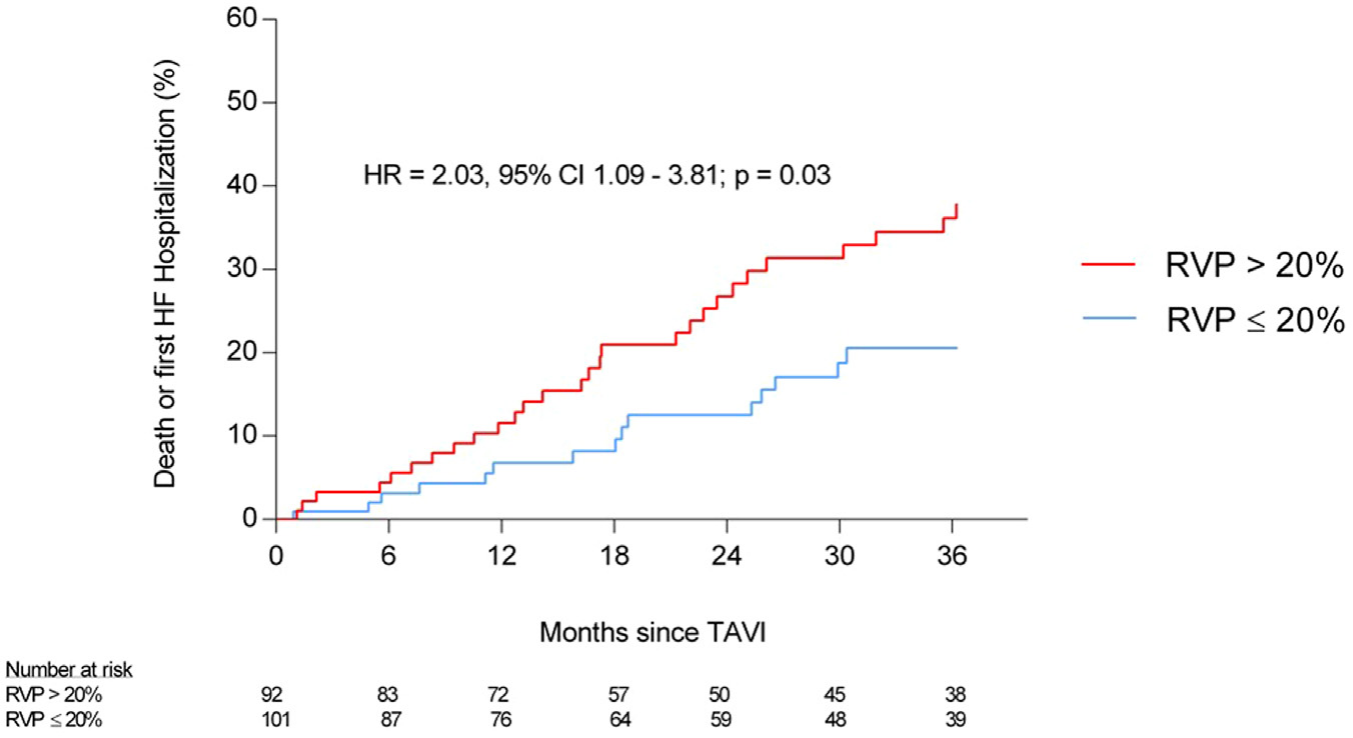
Incidence of death or HF hospitalization according to RVP percentage. CI = confidence interval; HF = heart failure; HR= hazards ratio; RVP = right ventricular pacing; TAVI = transcatheter aortic valve implantation.

During a median follow-up of 27.7 months (IQR 12.2–36.6 months), 43 patients (22.3%) experienced the composite endpoint of all-cause mortality or first hospitalization for HF. This included 28 of 92 patients (30.4%) in the > 20% VP group and 15 of 101 patients (14.9%) in the ≤ 20% VP group. A higher RVP burden (> 20%) was significantly associated with an increased risk of the composite endpoint (HR 2.03, 95% CI 1.09–3.81, *P* = .03) (Figure 3). No other independent predictive factors for HFH or death were identified (Table 3). A first HFH occurred in 24 patients (12.4%), including 16 of 92 (17.4%) in the > 20% VP group and 8 of 101 (7.9%) in the ≤ 20% VP group. All-cause death was observed in 30 patients (15.5%), with 21 of 92 (22.8%) in the > 20% VP group and 9 of 101 (8.9%) in the ≤ 20% VP group.

**Table 3:** Predictors of heart failure hospitalization or death

Variables	Univariable HR (95% CI)	*P* value	Multivariable OR (95% CI)	*P* value
Age (y)	1.0 (0.95–1.05)	.86		
Sex (male)	1.24 (0.67–2.27)	.49		
BMI (kg/m^2^)	1.04 (0.98–1.10)	.20		
Valve-in-valve implant	0.89 (0.27–2.89)	.84		
LVEF	0.99 (0.96–1.01)	.31		
Respiratory failure	1.92 (0.92–4.0)	.083	1.74 (0.83–3.65)	.14
Coronary artery stenosis	1.03 (0.56–1.92)	.92		
Diabetes	1.38 (0.72–2.65)	.34		
Hypertension	2.01 (0.85–4.78)	.11		
Dyslipidemia	1.08 (0.58–2.0)	.81		
Active smoking	1.49 (0.64–3.44)	.36		
RVP of > 20%	2.03 (1.09–3.81)	.027	1.95 (1.04–3.66)	.039

BMI = body mass index; CI = confidence interval; HR = hazard ratio; LVEF = left ventricular ejection fraction; OR = odds ratio; RVP = right ventricular pacing.

**Figure 4:**
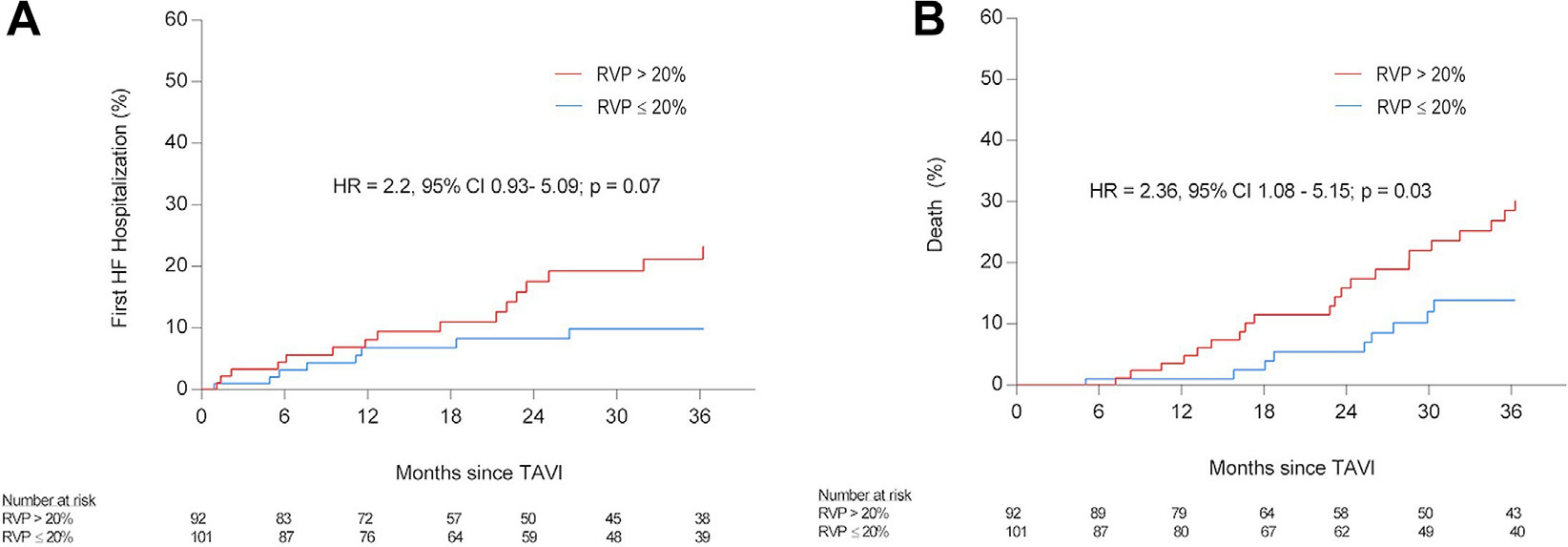
Incidence of HF hospitalization (**A**) and death (**B**) according to RVP percentage. CI = confidence interval; HF = heart failure; HR = hazards ratio; RVP = right ventricular pacing; TAVI = transcatheter aortic valve implantation.

An RVP burden of > 20% was significantly associated with a higher incidence of all-cause mortality (HR 2.36, 95% CI 1.08–5.15, *P* = .03), whereas its association with first HFH did not reach statistical significance (HR 2.2, 95% CI 0.93–5.09, *P* = .07) (Figure 4).

## Discussion

In this observational study performed in a large cohort of patients, male gender, AF, and high-degree AV block after the TAVR procedure were found to be independent predictors of a high burden of RVP during follow-up. The H_2_AS score, which incorporates these baseline clinical variables, could effectively predict the likelihood of high RVP rates in post-TAVR patients with PPI. A worse outcome was observed in patients with > 20% VP compared with those with ≤ 20% VP. Of importance, our study aimed not to investigate predictive factors for PPI indications after TAVR because only patients who had a validated indication for PPI according to current guidelines^13^ were included. Even a low rate of RVP remains clinically relevant in cases of paroxysmal AV block, and our risk score does not challenge the current indication for PPI.

To the best of our knowledge, this is the first study to investigate predictive factors for an RVP rate of > 20% specifically in post-TAVR PPI patients. This cutoff value is now widely used to define a high burden of VP, as underlined by the last consensus on CSP.^14^ By contrast, Bruno and colleagues^15^ used a higher cutoff value of 40% and identified independent predictors of high RVP burden such as LVEF, right bundle branch block, baseline QRS duration, type of prosthesis, and complete AV block. However, their study was not designed to identify predictive factors of RVP rate in post-TAVR PPI patients. In our study, all patients (except those in complete AV block at discharge) were intentionally programmed with algorithms promoting spontaneous AV conduction. In addition, pacemakers were reprogrammed with such algorithms at 3-month follow-up for patients demonstrating AV conduction recovery. This systematic approach ensured accurate assessment of predictive factors without bias. Permanent AF was a strong predictive factor for a high burden of RVP. In these patients, VP burden is largely determined by the choice of the lower rate limit, which was set at 60 beats per minute in our post-TAVR population with slowly conducted AF or AF with complete AV block. As expected, sinus node dysfunction was associated with a lower risk of having an RVP burden of > 20%. Interestingly, a reduced risk was also observed in patients implanted for LBBB associated with a prolonged HV interval (> 70 ms). Although these conduction abnormalities may indicate a predisposition to progression toward advanced AV block, our findings suggest that many of these patients remain below the 20% threshold of significant RVP burden during follow-up.

Pathophysiological studies have demonstrated that chronic apical RVP causes electrical dyssynchrony, leading to reduced cardiac output, impaired LVEF, increased pulmonary capillary pressure, and diastolic dysfunction.^16^, ^17^, ^18^ Initially, an RVP rate of > 40% was recognized as a significant risk factor for PICM (MOST trial).^19^ However, recent studies, such as those by Kiehl and colleagues,^11^ indicate that an RVP rate of > 20% already elevates the risk of PICM by 7-fold compared with rates of < 20%. Consistent with findings from the general population, the PACE-TAVI trial^15^ linked RVP of > 40% with poorer survival and higher HFH rates. Our study supports these observations and provides additional evidence of the adverse effects of chronic RVP, even at lower cutoff values.

Given the detrimental effects of pacing-induced dyssynchrony, CSP—which preserves left ventricular (LV) synchronization^20^—may offer significant benefits for patients with a high anticipated RVP burden. The study by Niu and colleagues^21^ highlighted the advantages of CSP in patients with high-degree AV block after TAVR, demonstrating improved LVEF and reduced LV end-diastolic diameter with CSP (left bundle branch area pacing [LBBAP] or His bundle pacing) compared with RVP. Although both His bundle pacing and LBBAP are feasible in this setting,^21^, ^22^, ^23^ LBBAP has demonstrated higher success rates, particularly in patients with self-expanding valves compared with balloon-expanding valves.^23^ For patients with low anticipated RVP burdens, leadless pacing may be preferable, offering reduced risks of long-term complications, such as infections or lead-related complications.^24^

Although CSP is now routinely performed and pacemaker programming aims to minimize unnecessary VP, the pacing site and device type can only be changed at the cost of reintervention. By predicting high RVP rates after PPI after TAVR, the H_2_AS score could serve as an important decision-making tool to guide the choice between CSP and leadless pacing, enabling personalized pacing strategies to optimize outcomes.

### Limitations

Although promising, the H_2_AS score requires validation in a larger, external population to ensure its broad applicability. As a single-center observational study, our findings may be subject to inherent biases. Only a few patients met the clinical composite endpoint, which may have limited the power of the secondary analysis. In addition, excluding patients followed up at other institutions may have introduced selection bias. Patients undergoing CSP were excluded, because they were typically programmed in DDD mode with a short AV delay to preserve or restore LV synchrony, resulting in an RVP burden close to 100%. This specific programming would have precluded the assessment of predictors of high RVP burden. Moreover, given that CSP preserves LV synchrony, its inclusion would likely have impacted the analysis of the composite clinical outcome.

## Conclusion

Male gender, permanent AF, and high-degree AV block as an indication for PPI were identified as independent predictors of high RVP burden in post-TAVR patients requiring PPI. The H_2_AS score, which integrates these clinical parameters, reliably predicts high RVP rates and can guide clinicians in choosing the most appropriate pacing strategy. Further studies are warranted to validate the H_2_AS score and assess the benefit of an individualized pacing strategy in post-TAVR patients.
